# Assessing the Real-Time Impact of COVID-19 on TB and HIV Services: The Experience and Response from Selected Health Facilities in Nairobi, Kenya

**DOI:** 10.3390/tropicalmed6020074

**Published:** 2021-05-10

**Authors:** Irene Mbithi, Pruthu Thekkur, Jeremiah Muhwa Chakaya, Elizabeth Onyango, Philip Owiti, Ngugi Catherine Njeri, Ajay M.V. Kumar, Srinath Satyanarayana, Hemant D. Shewade, Mohammed Khogali, Rony Zachariah, I. D. Rusen, Selma Dar Berger, Anthony D. Harries

**Affiliations:** 1 Respiratory Society of Kenya, Regent Court, Block A Suite A6 Hurlingham, Argwings Khodhek Road, Nairobi P.O. Box 43844-00100, Kenya; imbithi@resok.org (I.M.); chakaya.jm@gmail.com (J.M.C.); 2 International Union Against Tuberculosis and Lung Disease, 68 Boulevard Saint Michel, 75006 Paris, France; Pruthu.TK@theunion.org (P.T.); akumar@theunion.org (A.M.V.K.); SSrinath@theunion.org (S.S.); HShewade@theunion.org (H.D.S.); sberger@theunion.org (S.D.B.); 3 International Union Against Tuberculosis and Lung Disease, South-East Asia Office, C-6 Qutub Institutional Area, New Delhi 110016, India; 4 Department of Medicine, Therapeutics and Dermatology, Kenyatta University, Nairobi P.O. Box 43844-00100, Kenya; 5 Department of Clinical Sciences, Liverpool School of Tropical Medicine, Pembroke Place, Liverpool L3 5QA, UK; 6 Division of National TB, Leprosy and Lung Disease Programme, Ministry of Health, Afya House, Cathedral Road, Nairobi P.O. Box 30016-00100, Kenya; lizonyango2004@gmail.com (E.O.); philip.owiti@gmail.com (P.O.); 7 National AIDS and STDs Control Programme, Ministry of Health, Afya House, Cathedral Road, Nairobi P.O. Box 30016-00100, Kenya; Ncatherine26@gmail.com; 8 Yenepoya Medical College, Yenepoya (Deemed to be University), University Road, Deralakatte, Mangalore 575018, India; 9 Special Programme for Research and Training in Tropical Disease (TDR), World Health Organization, Avenue Appia 20, 1211 Geneva, Switzerland; khogalim@who.int (M.K.); zachariahr@who.int (R.Z.); 10 Vital Strategies, 100 Broadway 4th Floor, New York, NY 10005, USA; irusen@vitalstrategies.org; 11 Department of Clinical Research, Faculty of Infectious and Tropical Diseases, London School of Hygiene and Tropical Medicine, Keppel Street, London WC1E 7HT, UK

**Keywords:** COVID-19, Kenya, Nairobi, presumptive tuberculosis, tuberculosis, TB treatment outcomes, HIV, antiretroviral therapy, EpiCollect5, operational research

## Abstract

There was concern that the COVID-19 pandemic would adversely affect TB and HIV programme services in Kenya. We set up real-time monthly surveillance of TB and HIV activities in 18 health facilities in Nairobi so that interventions could be implemented to counteract anticipated declining trends. Aggregate data were collected and reported monthly to programme heads during the COVID-19 period (March 2020–February 2021) using EpiCollect5 and compared with monthly data collected during the pre-COVID period (March 2019–February 2020). During the COVID-19 period, there was an overall decrease in people with presumptive pulmonary TB (31.2%), diagnosed and registered with TB (28.0%) and in those tested for HIV (50.5%). Interventions to improve TB case detection and HIV testing were implemented from August 2020 and were associated with improvements in all parameters during the second six months of the COVID-19 period. During the COVID-19 period, there were small increases in TB treatment success (65.0% to 67.0%) and referral of HIV-positive persons to antiretroviral therapy (91.2% to 92.9%): this was more apparent in the second six months after interventions were implemented. Programmatic interventions were associated with improved case detection and treatment outcomes during the COVID-19 period, suggesting that monthly real-time surveillance is useful during unprecedented events.

## 1. Introduction

On 11 March 2020, the World Health Organization (WHO) declared a global pandemic of coronavirus disease 2019 (COVID-19), caused by a novel coronavirus named severe acute respiratory syndrome coronavirus 2 (SARS-CoV-2). By the end of 2020, nearly 80 million cases of COVID-19 and 1.8 million deaths had been reported globally to the organization [1]. In the first few months of the global pandemic, the epicenters of the pandemic were China, Europe and the USA, and there was concern that Africa, with its large volume of air traffic connections with these countries, would be the next region hit hard by COVID-19 [2,3].

At the beginning of the COVID-19 pandemic, political attention, healthcare workers, resources and finances were directed to the health sector to enable it to cope with the looming crisis. There was also quarantine, restricted movement and increased time spent indoors by the general population. All of this led to concerns that countries with high burdens of tuberculosis (TB) and human immunodeficiency virus/acquired immune deficiency syndrome (HIV/AIDS) might be unable to provide uninterrupted and quality healthcare services to their patients [4]. It was thought that health-seeking behavior and access to care for affected patients might also be adversely affected [5]. 

Similarities were drawn with the Ebola virus disease outbreak in Sierra Leone and Liberia in 2014, which took the two West African countries by surprise. Through restrictions in travel, “no-touch” policies and community fear of health facilities, the ability of the national TB programmes in these countries to diagnose TB and continue with HIV testing of TB patients was adversely affected, and, in the case of Liberia, treatment success rates in TB patients declined [6,7]. HIV testing capabilities for the general population decreased in both countries, although access to antiretroviral therapy (ART) was maintained [8,9]. Early on in the COVID-19 pandemic, The Stop TB Partnership and WHO issued guidance about how people with TB could protect themselves and how national TB programmes might adapt to the COVID-19 pandemic and national lockdowns [10,11]. UNAIDS provided similar advice to people living with HIV [12]. This global advice was augmented by urgent calls for practical planning to tackle the looming threat of COVID-19 in Africa [5,13]. 

We, therefore, set up a pilot project to measure the burden of COVID-19 and assess its impact on TB and HIV services in three Sub-Saharan African countries, Kenya, Malawi and Zimbabwe. This is the report from Kenya.

The first COVID-19 case reported to WHO by Kenya was on the 14 March 2020. By 15 April, Kenya had reported 216 COVID-19 cases (with nine deaths) [14]. How severe the COVID-19 storm would be, how long it would last and what impact it would have on public health services for TB and HIV/AIDS, was unknown. It was felt that being prepared, however, was key to being able to cope. Guinea in West Africa, for example, weathered the Ebola virus disease storm and managed to uphold TB services during this challenging period [15].

Relevant public health authorities in Kenya (including the National Tuberculosis, Leprosy and Lung Disease Programme (NTLD-P), the National AIDS & STI Control Programme (NASCOP) and the Respiratory Society of Kenya), working in close collaboration with the International Union Against Tuberculosis and Lung Disease (The Union), the Special Programme for Research and Training in Tropical Disease at WHO (TDR) and Vital Strategies (RESOLVE) therefore aimed at the early stage of the COVID-19 pandemic to strengthen the routine and real-time monitoring and evaluation system for TB and HIV case detection. The quarterly (every three months) recording and reporting system was strengthened in selected health facilities in the capital city of Nairobi by recording and reporting on TB and HIV parameters every month. We hypothesized that if there were decreases in TB case detection, diagnosis and treatment, and reductions in persons presenting for HIV testing or those diagnosed HIV-positive being referred for ART, then programmes could act more quickly on monthly information than quarterly information to try and reverse these trends. 

The overall aim of the study was to determine the impact of the COVID-19 pandemic on TB programme activities and HIV services through strengthened real-time surveillance in 18 selected health facilities in Nairobi, Kenya. The specific objectives were on a monthly basis to: (i) document the increase in nationally reported cases and deaths due to COVID-19 and its effects on general health services; (ii) collect, collate and report on specific TB and HIV-related parameters during the COVID-19 period (March 2020–February 2021), (iii) document the programmatic responses to changes in TB and HIV diagnosis and treatment during the COVID-19 period; and (iv) compare the findings with data collected and collated for the same TB and HIV parameters during the pre-COVID-19 period (March 2019–February 2020). 

## 2. Materials and Methods

### 2.1. Study Design

This was a cohort study using programmatically collected aggregate data. 

### 2.2. Setting

#### 2.2.1. General Setting: Kenya and Nairobi

Kenya is an East African country located along the Equator. The country is bordered by Somalia, Sudan, Ethiopia, Uganda and Tanzania. In 2019, the population was estimated at almost 53 million, with 32% living in urban areas, and life expectancy at birth was estimated at 66 years [16,17]. The major drivers of the country’s economy have been agriculture, fishing, forestry, education, retail trade, construction and financial services [16]. In 2019, the gross national income per capita was approximately USD 1750 [17]. 

Nairobi is the capital city and one of the 47 semi-autonomous counties in the country. In 2019, it had an estimated population of 4.4 million. The current study took place in the City County of Nairobi because nearly 80% of COVID-19 cases in Kenya were reported from this area at the onset of the outbreak. The County of Nairobi is divided into 10 TB control zones with just over 1000 registered health facilities [18]. To achieve good county geographical representation, two well-established facilities from each of these 10 TB control zones were purposively selected in consultation with the head of the TB Programme and the County TB and Leprosy coordinator. Selected facilities had the following on-site characteristics: TB diagnostic capability (smear microscopy and/or the Xpert MTB/RIF^®^ assay); TB treatment and monitoring services; HIV testing and ART referral or treatment services. 

The initial sites included seven hospitals, nine health centers and four dispensaries. Two of these health facilities had to be excluded before starting. One health center on the Kenyatta University Campus closed at the start of lockdown and remained closed indefinitely thereafter. One of the hospitals required its own ethics approval for the study. However, this was going to take too long and the hospital had to be excluded. This left 18 health facilities in total available for the duration of the study. The established staff who were already working in these facilities delivering TB and HIV services helped with the monthly collection of data. 

#### 2.2.2. TB and HIV Services

The diagnosis and treatment of TB and HIV/AIDS in Kenya are the responsibility of the Ministry of Health. People with symptoms suggestive of TB (cough, fever, weight loss and night sweats) are classified as having presumptive pulmonary TB (PTB). They are recorded as such when attending a health facility, along with their demographic details. Investigations are carried out according to national guidelines [19], using sputum smear microscopy and/or the Xpert MTB/RIF^®^ assay to establish a bacteriologically confirmed diagnosis of PTB. In the other patients, clinical assessment, radiography and other circumstantial evidence are used to establish a diagnosis of clinically diagnosed PTB or extrapulmonary TB (EPTB). Patients with diagnosed TB (drug-susceptible and drug-resistant) are registered and started on anti-TB treatment in line with national and international guidelines [19,20]. Treatment outcomes are monitored, recorded and reported according to international guidelines [21]. 

HIV testing is institutionalized in the public health facilities and routinely offered to anyone attending for care and also to TB patients according to national and international guidelines [22,23]. HIV testing is carried out using rapid testing algorithms. Those diagnosed HIV-positive are referred to ART services for immediate start of ART regardless of WHO clinical stage or CD4 cell count.

There is generally good quality data capture and reporting for TB and HIV/AIDS at all levels due to regular supervision by county and national programme supervisors.

#### 2.2.3. Data Monitoring, Recording and Reporting 

For this study, we selected only patients with drug-susceptible TB who were treated and monitored with the standard six month regimen [19,20]. Data were routinely collected on a daily basis by healthcare workers in each study site using the standard existing monitoring tools. These included: the TB presumptive and/or the TB laboratory register for presumptive TB depending on the facility; the TB patient register for those diagnosed and registered with TB and in which TB treatment outcomes were also recorded; and the HIV Testing Services (HTS) Register in which those diagnosed HIV-positive and referred for ART were recorded. These registers were mainly paper-based. One to two weeks after the end of each month, the project country coordinator (IM—who was appointed specifically for the study) visited each site along with her team of trained data collectors. They collated the individual data on TB and HIV variables for the previous month into monthly aggregate data, which they then entered into a data form developed using an EpiCollect5 mobile application (https://five.epicollect.net, accessed on 19 April 2021). 

For TB treatment outcomes, we took the monthly cohorts of patients who had been enrolled onto treatment eight months previously – this allowed for six months of treatment to be completed and a further two months for outcomes to be validated and documented in the records. Thus, for example, the August 2020 TB treatment outcome data were obtained for the TB patients enrolled and started on treatment in January 2020. National data on COVID-19 cases and deaths reported to WHO on the last day of the month were obtained from WHO situation and epidemiological reports [1]. When collecting the monthly data during the COVID-19 period, the same procedures were used to collect data for the same month one year previously (termed the pre-COVID-19 period). 

Once all the data for the month had been entered into EpiCollect5, they were checked and validated by the project country coordinator and the overall project monitoring and evaluation officer (PT) based at The Union. Data were then presented in a monthly report to the heads of the NTLD-P and NASCOP and all other relevant stakeholders involved in the project. Key policy or practice changes made at the local facility, county, or national level during that month to explain and/or counteract the effects of COVID-19 on TB and HIV parameters were documented in a narrative table within the report. These monthly reports always reached the national programme staff heads within four weeks of closure of that month to enable timely surveillance and possible action.

### 2.3. Study Population

The study population included all patients presenting to TB services with presumptive TB, all TB patients registered for TB treatment and all persons tested for HIV in 18 health facilities in Nairobi, Kenya, between March 2019 and February 2021: the COVID-19 period was designated as March 2020 to February 2021, and the pre-COVID-19 period was designated as March 2019 to February 2020. For assessment of TB treatment outcomes, we included TB patients who started on treatment from (i) August 2018 to July 2019 (pre-COVID-19 cohort) and (ii) August 2019 to July 2020 (COVID-19 cohort).

### 2.4. Data Variables, Sources of Data and Timing of Data Collection 

Data variables for TB included aggregate numbers of presumptive PTB patients, stratified by male and female and adults (≥15 years) and children (<15 years); presumptive PTB patients who were diagnosed bacteriologically positive by either smear microscopy and/or Xpert MTB/RIF^®^; registered TB patients, stratified by bacteriologically confirmed PTB, clinically diagnosed PTB and EPTB; registered TB patients who were newly tested for HIV in that month after being diagnosed with TB—this excluded patients who already knew they were HIV-positive; standardized TB treatment outcomes of those patients enrolled for treatment eight months previously–these outcomes included treatment success (a combination of those cured and those who completed treatment with no sputum smear examination), lost to follow-up (LTFU), died, failed treatment or not evaluated [21]. Not evaluated is an outcome given to those who transfer from one facility to another and for whom the final treatment outcome is not recorded. Data variables for HIV included an aggregate number of persons who were HIV tested, stratified by male and female and adults (≥15 years) and children (<15 years); persons diagnosed HIV-positive; HIV-positive persons referred to ART services. 

Sources of primary individual data were the TB presumptive and/or TB laboratory register, the TB patient register, and the HTS register. The national COVID-19 cases and deaths reported to WHO on the last day of the month were obtained from WHO situation and epidemiological reports [1]. Aggregate data were collected from the primary data sources and uploaded to an EpiCollect5 application, where they were checked and validated, and this was carried out between June 2020 and March 2021.

### 2.5. Analysis and Statistics 

Aggregate data were presented as frequencies and proportions, and comparisons were made between the COVID-19 period and the pre-COVID-19 period. The percentage difference (decline or increase) in numbers during each month of the COVID-19 period was calculated relative to the numbers during the same month of the pre-COVID-19 period. The relative percentage differences observed between the first six months of COVID-19 (March to August 2020) and the second six months of COVID-19 (September 2020 to February 2021) were also calculated.

## 3. Results

### 3.1. COVID-19 Cases, Deaths and General Effects on Health Services

**Figure 1:**
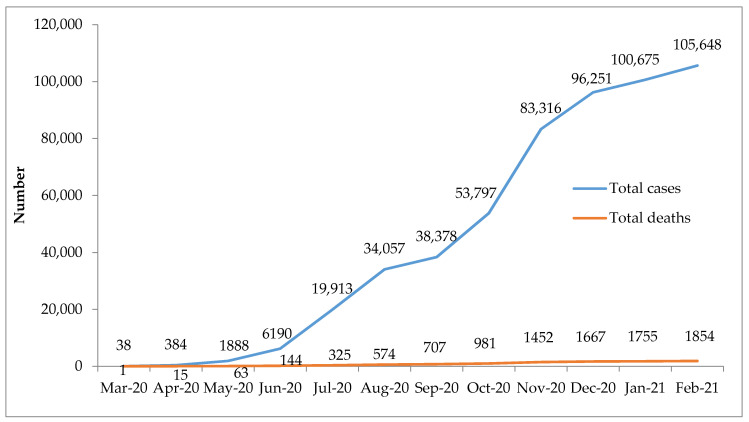
Cumulative number of COVID-19 cases and deaths in Kenya between March 2020 and February 2021, as reported to the World Health Organization.

There was a gradual increase in COVID-19 cases and deaths during the 12 months, with 105,648 cases and 1,854 deaths reported to WHO by the end of February 2021 (Figure 1). 

In terms of general effects on services, there was a national lockdown between March 2020 and the end of June 2020. The lockdown resulted in enforced travel restrictions, shorter working hours and intermittent closures of health facilities (due to lack of personal protective equipment and sickness of healthcare workers from COVID-19). Many staff, including those working in TB and HIV, were repurposed for COVID-19 work. After the period of lockdown, there was a widespread strike by health workers, many of whom refused to come to work from November 2020 to January 2021. This resulted in some health facilities having to temporarily close down or partially close down again. There had also been previous widespread strike action in the health sector in the pre-COVID-19 period from July to October 2019. Finally, there was widespread fear and stigma about COVID-19 in the community, with people reluctant to visit health facilities due to fear of contracting COVID-19 and being diagnosed with the disease.

### 3.2. TB Case Finding, Diagnosis and Registration 

There was an overall decrease in the aggregate numbers of persons presenting with presumptive PTB and being diagnosed and registered with TB in the COVID-19 period when compared to the pre-COVID-19 period (Table 1).

For presumptive PTB, the overall decrease was greater in children (50.2%) than in adults (27.3%) and greater in females (35.2%) than in males (26.0%). The yield of bacteriologically positive PTB in those investigated for presumptive TB increased (0.1%) marginally. For registered TB, the overall decrease was greater in bacteriologically confirmed PTB and was similar for the other two types (clinically diagnosed PTB and EPTB). The percentage of patients who were newly HIV tested out of those eligible for testing slightly decreased (1.7%).

**Figure 2:**
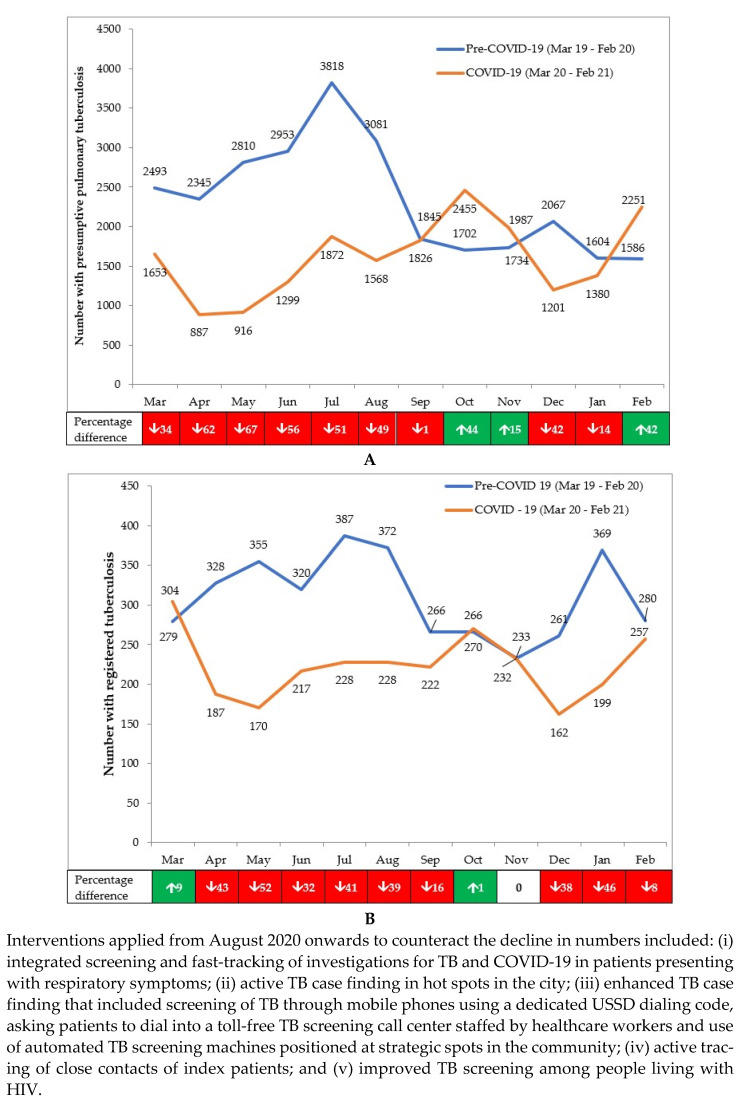
(**A**) Numbers presenting each month with presumptive PTB in 18 health facilities in Nairobi, Kenya, during pre-COVID-19 and COVID-19 periods (**B**) Numbers presenting each month with registered TB in 18 health facilities in Nairobi, Kenya, during pre-COVID-19 and COVID-19 periods.

The monthly number of people presenting with presumptive PTB and those registered for TB in the pre-COVID-19 and COVID-19 periods are shown in Figure 2A,B. The footnotes in the figures indicate the interventions put in place from August 2020 onwards to counteract the downward trends. Compared with the pre-COVID-19 period, the decline in presumptive TB in the first six months of the COVID-19 pandemic (March to August 2020) was 53.2%, which was very different to the 5.2% increase observed in the second six months (September 2020 to February 2021). The decline in registered TB in the first six months of the COVID-19 pandemic was 34.7%, which was greater than the 19.9% decrease observed in the second six months.

### 3.3. TB Treatment Outcomes 

The overall aggregate treatment outcomes between the pre-COVID-19 and COVID-19 periods are shown in Table 2. There was a slight increase in treatment success in the COVID-19 period (2.0%), mainly due to an overall decrease in patients “not evaluated” (2.2%). Other adverse programme outcomes were similar between the two periods. 

**Figure 3:**
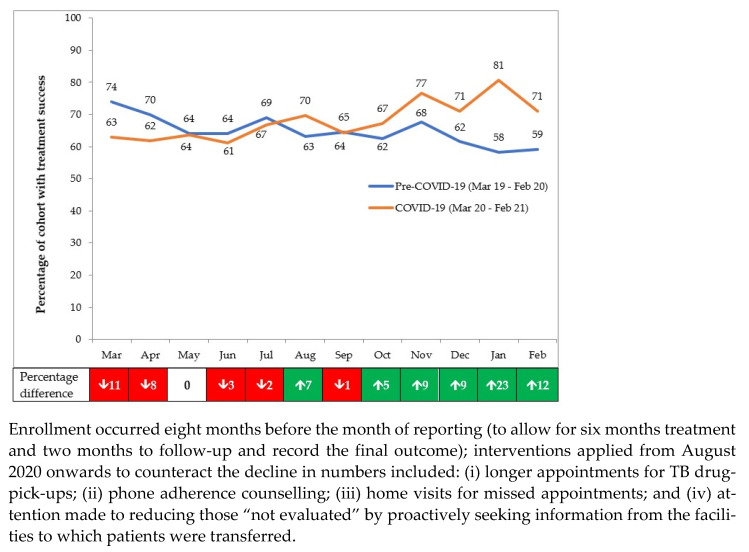
Treatment success among those enrolled each month in 18 health facilities in Nairobi, Kenya, during pre-COVID-19 and COVID-19 periods.

The monthly treatment success rates in the pre-COVID-19 and COVID-19 periods are shown in Figure 3. The footnotes indicate the interventions put in place from August 2020 onwards to counteract the downward trends. Compared with the pre-COVID-19 period, the decline in treatment success in the first six months of COVID-19 was 2.5%, which was very different to the 9.7% increase observed in the second six months.

### 3.4. HIV Testing Among Those Visiting the Health Facilities and Referral to ART

There was an overall aggregate decrease in numbers tested for HIV between the pre-COVID-19 and COVID-19 periods (see Table 3). The overall decrease was greater in adults (50.8%) than in children (43.0%) and almost the same between males (51.2%) and females (50.2%). There was a slight increase in the HIV-positivity rate (1.1%), and the proportion of those HIV-positive referred to ART (1.7%) in the COVID-19 period. 

**Figure 4:**
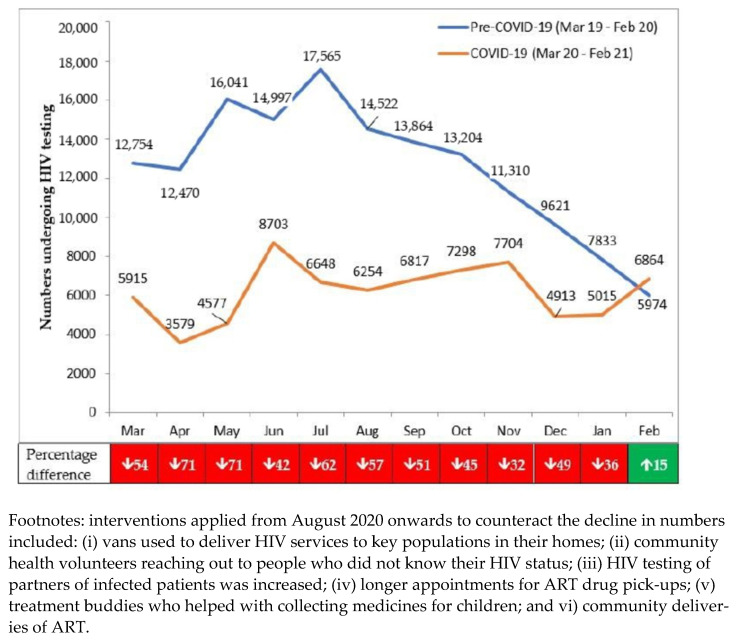
Numbers presenting each month for HIV testing in 18 health facilities in Nairobi, Kenya, during pre-COVID-19 and COVID-19 periods.

The monthly numbers tested for HIV in the pre-COVID-19 and COVID-19 periods are shown in Figure 4. Numbers were already declining in the pre-COVID period due to several factors that included: promotion of HIV self-testing, with the referral of only those HIV-positive to the health facilities for confirmation of the result; a large number of people already tested in the capital city through various testing campaigns; and more targeted testing of high-risk groups. The decline in HIV testing stabilized once COVID-19 appeared. The more targeted testing strategies and confirmation of HIV self-testing results might have explained the increase in HIV positivity from March 2020 onwards. 

The HIV/AIDS programme implemented several interventions from August 2020 onwards to counteract the low numbers presenting for HIV testing (see Figure 4, footnotes). Compared with the pre-COVID period, the decline in HIV-testing in the first six months of COVID-19 was 59.6%, which was greater than the 39.8% decline observed in the second six months. 

## 4. Discussion

This is the first study in Nairobi, Kenya, to compare TB case detection, TB treatment outcomes, HIV testing and referral to ART on a month-by-month basis between the COVID-19 and pre-COVID-19 periods. There were three important findings, which require explanation and interpretation. 

First, with respect to TB programme activities, there was considerable variation in TB case detection numbers during the pre-COVID-19 period. Numbers with presumptive PTB increased between April and July 2019 (the summer months), and this is in line with what usually happens each year at this particular time, both at national level and in the city county of Nairobi (source: NTLD-P). From July to October 2019, however, there was widespread strike action among the healthcare workers which severely affected health services, including TB case finding which declined dramatically. When the strike action was over, numbers with presumptive TB did not increase. There were two possible reasons for this that included (i) TB case numbers between 2018 and 2019 already being in decline, and this may have been part of the process, and (ii) community confidence in the health sector being adversely affected and taking time to pick up. In contrast, numbers with registered TB did increase, showing that the health services had returned to some degree of normality. 

In spite of these pre-COVID-19 variations, there was still an overall significant decline in numbers presenting to the health facilities with presumptive PTB and in numbers being diagnosed and registered with TB in the COVID-19 period. The expected upward trend in TB case finding from April to July 2020 (the summer months) did not happen. Children and women were particularly affected, maybe because there was more difficulty for mothers and children to move around during lockdown and a heightened sense of fear for the family about accessing health facilities. The national lockdown and the ongoing challenges after lockdown, which included health facility closures and strike action in the health sector, posed ongoing problems. While numbers with presumptive TB picked up in the second six months of the COVID-19 period and particularly in the last month of the study, numbers with registered TB remained below pre-COVID-19 levels throughout this time. 

These findings of declines in TB case detection are similar to reports from other health facilities and clinics in Nigeria [24], Brazil [25], China [26] and India [27]. Our study and these clinic reports align with a recent report from WHO showing an overall 21% shortfall in TB case notifications in 84 countries in 2020 compared with 2019 [28]. A modelling analysis at the start of the pandemic suggested that a three month suspension of TB services due to COVID-19 lockdown followed by ten months restoration back to normal would cause over five years an additional 25,000 TB cases and 12,500 TB deaths in Kenya, mainly as a result of the accumulation of undetected TB during lockdown [29]. A further modelling study in high-burden, low- and middle-income countries predicted a 20% increase in TB mortality, the greatest impact coming from reductions in timely diagnosis and treatment of new TB cases [30]. However, these modelling studies were done early on in the pandemic when there was hope that service disruption would be temporary. The reality is that service disruption has continued throughout the year, and has been further exacerbated by industrial action in the health sector, health facility closures and continued community fear and stigma about COVID-19. 

The city of Nairobi, therefore, has done well to introduce an array of innovative measures such as integrated screening for TB and COVID-19, TB self-testing, active case finding and contact tracing to mitigate the challenges. These seem to have paid off with improvements in case detection and diagnosis in the later months of the COVID-19 period. The measures undertaken in Nairobi are in line with those recently recommended by the Stop TB Partnership [31], which include screening patients with respiratory symptoms for both TB and COVID-19, creating, developing and supporting networks of TB survivors and TB communities and implementing real-time surveillance data.

Second, it was encouraging to see that while TB treatment success initially decreased, it then picked up to between 70% and 80% in the last four months of the study. At the start of the study, we were concerned that COVID-19 restrictions would prevent patients from collecting anti-TB medications, compromise drug adherence and reduce the ability of TB programme staff from obtaining information about final treatment outcomes. TB patients with associated COVID-19 coinfection have an increased risk of mortality [32,33]. We were worried that undetected COVID-19 in our TB patients might increase TB deaths during treatment. In the event, however, there was a minimal increase in the risk of death. The TB programme implemented patient-centered measures to help patients comply with treatment and adhere to medication, and attention was paid to reducing the “not evaluated” treatment outcome. All these measures were associated with improvements in treatment success.

Third, with respect to HIV services, there was already a marked decline in HIV testing numbers at the health facilities in the pre-COVID-19 period. While the health sector strike action between July and October 2019 played a part, there were additional explanations. HIV testing had been institutionalized in public health facilities over the years and many people visiting these facilities had already been HIV tested. NASCOP had started promoting HIV-self testing, only doing confirmatory tests in health facilities for those who had tested HIV-positive. The country was also moving towards a more targeted HIV testing approach directed at high-risk groups as recommended for Sub-Saharan Africa as a whole and for Kenya [34,35]. 

Despite the pre-COVID-19 decline, HIV testing numbers in health facilities remained low during the 12 months of COVID-19. The reduction in numbers is similar to the reductions in HIV testing volumes that have been documented in Europe, the USA and other countries in Africa [36,37,38,39]. The decrease in HIV testing threatens access to diagnosis and treatment, resulting in excess HIV-related deaths and ongoing transmission of HIV in the community. HIV testing started to improve in the second six months, perhaps due to implementing community outreach services for clients and the promotion of assisted partner testing as recommended nationally [40]. It was encouraging to see that referrals to ART were maintained above 90% over the 12 months, with a slight increase observed during the COVID-19 period.

There were several strengths to this study. First, we embedded monthly surveillance within the routine services of the health facilities. Second, there was cross-checking and validation of monthly data between the country coordinator and the overall study monitoring and evaluation officer. Third, we used two 12-month periods to compare data, which enabled us to account for any seasonal changes that might affect access to health facilities. Finally, the conduct and reporting of the study were in line with the Strengthening the Reporting of Observational Studies in Epidemiology (STROBE) guidelines [41].

There were, however, some limitations. Our study was limited to health facilities in Nairobi, and therefore may not be representative of Kenya as a whole. The use of aggregate data meant it was impossible to fully understand the individual cascade of care for TB case detection and HIV testing. We only assessed referral to ART and did not measure initiation and retention on ART. Previous studies have suggested that ART interruption has been a problem during the COVID-19 pandemic [42], and it would have been interesting to assess this in Kenya. We also did not assess treatment outcomes of patients with drug-resistant TB, but these patients would have been referred to other centers where the ability to track outcomes might have been difficult with COVID-19 restrictions in place. Finally, the official monthly reports to WHO of COVID-19 cases and deaths may have underestimated the actual burden, and therefore impact, of COVID-19 in the country. A seroprevalence study of anti-SARS-CoV-2 antibodies in Kenya between April and June 2020 estimated a crude national seroprevalence of 5.6%, with levels being highest in the counties of Mombasa (8.0%) and Nairobi (7.3%) [43]. A study on deceased people at the University Teaching Hospital morgue in Lusaka, Zambia, found that only 6 (9%) of 70 people, who had SARS-CoV-2 confirmed from postmortem nasopharyngeal swabs within 48 hours of death, had ever been tested before death [44]. These data suggest that there are many unreported cases and deaths due to COVID-19, a situation likely to be present in many other African countries.

Despite these limitations, there are some important programmatic implications from this study. First, the strengthened monthly surveillance system worked well with the disease control programme heads appreciating and closely reviewing the monthly reports. Although cause and effect are difficult to infer with this type of study, the frequent access to data may have helped with the implementation of interventions on the ground to counteract declining trends in TB case detection and treatment outcomes and HIV testing. However, to continue with this system requires funding and resources, which would need to be obtained. 

Second, with COVID-19 likely to become endemic, TB and COVID-19 care and treatment programmes need to think about further integration with respect to screening, laboratory infrastructure and diagnosis, contact tracing and sound infection, prevention and control measures, especially in health facility settings [45]. Resources permitting, more consideration should be given to the use of digital platforms to facilitate case finding and treatment [46]. Kenya also has a large private sector, which in the case of TB serves 20% of all patients registered and treated in the country [47], and this must not be forgotten when it comes to integration and innovation. HIV self-testing would undoubtedly fill a significant gap in clinic-based HIV testing at a time of crisis such as this [34,35], but more attention needs to be paid at the health facility level to recording and reporting on numbers self-tested along with the results.

Finally, research into improving the responses to TB and HIV/AIDS amidst the COVID-19 pandemic must continue [48]. This needs to be mainly focused on safely and effectively delivering the key programme activities and ensuring that patients can more easily access diagnostic, treatment and prevention services.

## 5. Conclusions

Using strengthened monthly real-time surveillance in 18 health facilities in Nairobi, Kenya, the number of people with presumptive TB and registered TB, as well as the number of people presenting for HIV testing, declined during 12 months of the COVID-19 outbreak compared with 12 months pre-COVID-19. Successful TB treatment and referral of HIV-positive persons to ART increased slightly during the COVID-19 period. Programmatic interventions on the ground were associated with an improvement in case detection and treatment outcomes in the second six months of the COVID-19 period compared to the first six months. This study strongly suggests that real-time operational research can generate useful evidence for real-time action.

## Figures and Tables

**Table 1:** Characteristics of persons with presumptive pulmonary TB and registered TB in 18 health facilities in Nairobi, Kenya, during pre-COVID-19 and COVID-19 periods.

Characteristics	Pre-COVID-19 Mar 2019–Feb 2020 N	COVID-19 Mar 2020–Feb 2021 n	Difference between Pre-COVID-19 and COVID-19 %
Presumptive Pulmonary TB (total)	28,038	19,295	↓31.2
Adults (≥15 years)	23,264	16,917	↓27.3
Children (<15 years)	4774	2378	↓50.2
Male	12,221	9043	↓26.0
Female	15,817	10,252	↓35.2
Bacteriologically positive	2221	1411	↓30.2
Positivity Rate (%)	(7.2%)	(7.3%)	↑0.1% *
Registered TB (Total)	3716	2676	↓28.0
Bacteriologically confirmed PTB	1985	1335	↓32.7
Clinically diagnosed PTB	1073	836	↓22.1
Extrapulmonary TB	658	505	↓23.3
Eligible for being newly HIV tested	3067	2259	↓26.3
Newly tested for HIV (%)	(94.7%)	(93.0%)	↓1.7% *

* absolute change (increase or decrease); TB = tuberculosis; PTB = pulmonary tuberculosis.

**Table 2:** Treatment outcomes in TB patients enrolled for treatment in 18 health facilities in Nairobi, Kenya, during pre-COVID-19 and COVID-19 periods.

Treatment Outcomes in Patients Enrolled for TB Treatment	Pre-COVID-19 Mar 2019–Feb 2020 n	COVID-19 Mar 2020–Feb 2021 n	Difference between Pre-COVID-19 and COVID-19 %
Enrolled for treatment:	3640	2366	
Treatment Success (%)	(65.0)	(67.0)	↑2.0 *
Loss to Follow-up (%)	(7.3)	(7.0)	↓0.3 *
Died (%)	(4.2)	(5.0)	↑0.8 *
Failed treatment (%)	(0.8)	(0.5)	↓0.3 *
Not evaluated (%)	(22.7)	(20.5)	↓2.2 *

* absolute change (increase or decrease); TB = tuberculosis; treatment outcome was considered “treatment success” when the TB patient was either cured or had “treatment completed”. The success rate was calculated for the month-wise cohort of TB patients commenced on treatment eight months before the reporting month (considering six months of treatment to be completed and another two months to finalize the recording of outcomes).

**Table 3:** Characteristics of persons tested for HIV and referred for antiretroviral therapy in 18 health facilities in Nairobi, Kenya, during pre-COVID-19 and COVID-19 periods.

Characteristics	Pre-COVID-19 Mar 2019–Feb 2020 n	COVID-19 Mar 2020–Feb 2021 N	Difference between pre-COVID-19 and COVID-19 %
People tested for HIV (Total):	150,155	74,287	↓50.5
Adults (≥15 years)	145,040	71,374	↓50.8
Children (<15 years)	5115	2913	↓43.0
Male	48,546	23,698	↓51.2
Female	101,609	50,589	↓50.2
Positive for HIV	3819	2673	↓30.0
HIV-Positivity Rate (%)	(2.5%)	(3.6%)	↑1.1 *
HIV-positive persons referred to ART (%)	(91.2%)	(92.9%)	↑1.7 *

* absolute change (increase or decrease); ART = antiretroviral therapy.

## Data Availability

The data that support the findings of the study are available from one of the first authors (P.T.) upon reasonable request.
